# GABA System Modifications During Periods of Hormonal Flux Across the Female Lifespan

**DOI:** 10.3389/fnbeh.2022.802530

**Published:** 2022-06-16

**Authors:** Rachel A. Gilfarb, Benedetta Leuner

**Affiliations:** ^1^Neuroscience Graduate Program, The Ohio State University, Columbus, OH, United States; ^2^Department of Psychology, The Ohio State University, Columbus, OH, United States; ^3^Department of Neuroscience, The Ohio State University, Columbus, OH, United States

**Keywords:** GABA, estradiol, progesterone, puberty, ovarian cycle, pregnancy, postpartum, menopause

## Abstract

The female lifespan is marked by periods of dramatic hormonal fluctuation. Changes in the ovarian hormones estradiol and progesterone, in addition to the progesterone metabolite allopregnanolone, are among the most significant and have been shown to have widespread effects on the brain. This review summarizes current understanding of alterations that occur within the GABA system during the major hormonal transition periods of puberty, the ovarian cycle, pregnancy and the postpartum period, as well as reproductive aging. The functional impacts of altered inhibitory activity during these times are also discussed. Lastly, avenues for future research are identified, which, if pursued, can broaden understanding of the GABA system in the female brain and potentially lead to better treatments for women experiencing changes in brain function at each of these hormonal transition periods.

## Introduction

The female lifespan is marked by periods of dramatic hormonal flux. The first of these periods is puberty, which is when the ovaries begin to secrete increasing amounts of estrogens and progestins (Jost et al., 1973), and when the ovarian cycle emerges in spontaneously ovulating species, such as humans, mice, and rats (Herbison, 2016, 2020). Fluctuations in 17β-estradiol (e.g., estradiol or E2), the predominant circulating estrogen in females, and progesterone, the main progestin, continue with each ovarian cycle until either: 1) pregnancy, when high levels of estradiol and progesterone are sustained for an extended period of time, followed by a precipitous drop at birth (Stewart et al., 1993; Tal and Taylor, 2000; Nair et al., 2017), or 2) the menopausal transition, when steep declines in ovarian hormones mark reproductive senescence (Wise, 1999; Genazzani et al., 2005; Nappi and Cucinella, 2020). These times are also accompanied by shifts in the neuroactive progesterone metabolite allopregnanolone (also known as 3α-Hydroxy-5α-pregnan-20-one, 3α,5α-Tetrahydroprogesterone, 3α,5α-THP, or ALLO).

The brain is highly responsive to changes in estradiol, progesterone, and ALLO, resulting in heightened plasticity during periods of hormonal flux (Sisk and Foster, 2004; Shanmugan and Epperson, 2014; Juraska and Willing, 2017; Piekarski et al., 2017b; Barrientos et al., 2019; Duarte-Guterman et al., 2019). Among the systems which exhibit plasticity across such periods is the GABA (γ-aminobutyric acid) system, which traffics the principal inhibitory neurotransmitter GABA (Shen et al., 2007; Smith et al., 2009; Kilb, 2012; Smith, 2013). Activity of the GABAergic system changes during hormonal transition periods due to the actions of ovarian hormones and their metabolites such as ALLO (Maguire and Mody, 2008; Smith, 2013; MacKenzie and Maguire, 2014; Wang et al., 2016, 2019). During times of hormonal change, adaptations of the GABA system are necessary to maintain excitatory and inhibitory balance (E/I balance). Failure to regulate E/I balance has been linked to changes in cognitive functioning, mood alterations, and susceptibility to the development of psychiatric disorders (Scharfman and MacLusky, 2006; Smith, 2013; MacKenzie and Maguire, 2014; Page and Coutellier, 2018; Sohal and Rubenstein, 2019).

**FIGURE 1:**
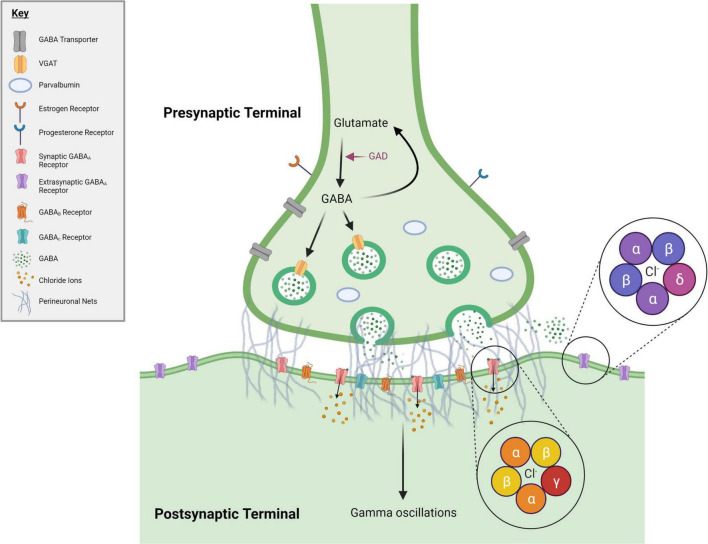
*GABAergic synapse.* During periods of hormonal flux across the female lifespan, numerous changes to the GABA system occur. Many of these changes occur within GABA synapses, represented here. Included in this figure are the elements of the GABA system discussed in this manuscript. Pink text denotes an enzyme. GABA = gamma-Aminobutyric acid, GAD = glutamate decarboxylase, VGAT = vesicular GABA transporter. Created with BioRender.com.

The GABA system consists of multiple components that together modulate inhibitory tone and aid in maintaining E/I balance (Figure 1). The GABA system includes GABA neurons, which are largely inhibitory interneurons expressing the proteins parvalbumin (PV), somatostatin (SST), or vasointestinal peptide (VIP) (Rudy et al., 2011; Tremblay et al., 2016). GABA neurons synapse on pyramidal neurons or each other to form a network in which GABA neurons control neural output (Meyer et al., 2011; Buzsáki and Wang, 2012). Resulting neural outputs synchronize and contribute to the production of gamma oscillations, which further influence the activity of surrounding neurons (Buzsáki and Wang, 2012). The GABAergic system is also comprised of perineuronal nets (PNNs), which primarily (though not exclusively) surround PV neurons and regulate the formation of synaptic connections to affect inhibitory gain (Karetko and Skangiel-Kramska, 2009; Bosiacki et al., 2019). GABA neurons and PNNs are hormonally sensitive (Hart et al., 2001; Blurton-Jones and Tuszynski, 2002; Maguire and Mody, 2008; Smith et al., 2009; MacKenzie and Maguire, 2014; Wu et al., 2014; Równiak, 2017; Drzewiecki et al., 2020; Uriarte et al., 2020), making them prime targets during periods of hormonal flux throughout the female lifespan. GABA_A_ receptors (GABA_A_Rs) are another facet of the GABA system that can modulate inhibition within the neurons that they populate (MacKenzie and Maguire, 2014; Braden et al., 2015). Synaptic and extrasynaptic GABA_A_Rs are ionotropic, pentameric receptors containing different subunits [α(1–6), β(1–4), γ(1–3), δ, ε, θ, π and ρ(1–3)] that create pores through which Cl- ion gradients form (Maguire and Mody, 2009). α and β subunits are present in all different receptor compositions; the γ subunit is mainly associated with GABA_A_Rs expressed in the synaptic compartment and mediates “phasic” inhibition, while the δ subunit is associated with extrasynaptic receptors and mediates “tonic” inhibition (Licheri et al., 2015). GABA_A_Rs are hormonally responsive, thus they can further influence inhibition within the female brain during times of hormonal change (Maguire and Mody, 2008; Smith et al., 2009; MacKenzie and Maguire, 2014). For example, ALLO can act as a positive allosteric modulator (PAM) at GABA_A_Rs to potentiate inhibitory activity and alter inhibitory tone (MacKenzie and Maguire, 2014).

This review explores how fluctuations in estradiol, progesterone, and ALLO promote alterations within the GABA system of the female brain during puberty, the ovarian cycle, pregnancy and the postpartum period, as well as reproductive senescence considering data from both humans and rodents. Additionally, this review discusses the functional consequences of altered inhibitory activity and suggests avenues for future research. Broadening our understanding of the GABA system across the female lifespan has important implications for mood regulation and cognition, which are also known to be modified during periods of hormonal flux (Maguire and Mody, 2009; MacKenzie and Maguire, 2014; Wu et al., 2014). Ultimately, expanding what is known about GABA system alterations during hormonal transition periods may further aid in the development of targeted strategies to maintain E/I balance and could lead to better treatments for women experiencing changes in brain function during these times.

## Puberty

Puberty, the transition to reproductive maturity, is a developmental stage when the ovaries begin to secrete increasing amounts of estrogens and progestins due to a rise in activity of the hypothalamic-pituitary-ovarian (HPO) axis (Jost et al., 1973; Keating et al., 2019; Delevich et al., 2021). In addition, ALLO gradually increases before and rapidly drops off with pubertal onset in rodents and humans (Fadalti et al., 1999; McCartney et al., 2007; Shen et al., 2007; Smith, 2013). Some evidence suggests that changes in estradiol, progesterone, and ALLO during puberty may play a key role in brain and behavioral maturation by altering inhibitory tone (Schulz et al., 2009; Holder and Blaustein, 2014; Jones and Lopez, 2014; Juraska and Willing, 2017; Delevich et al., 2021).

### Changes to the Gamma-Aminobutyric Acid System Following Pubertal Onset in Young Women

Adolescence, the developmental period beginning with pubertal onset, is characterized by protracted development of inhibition within late-developing brain regions like the prefrontal cortex (PFC) (Chugani et al., 2001; Fung et al., 2010; Silveri et al., 2013; Laube et al., 2020). Various changes within the human GABA system likely contribute to increases in inhibition over the course of adolescence. For example, cortical expression of the enzyme responsible for GABA synthesis (glutamate acid decarboxylase, GAD) peaks around puberty (Pinto et al., 2010). In addition, shifts in GABA_A_R subunit expression occur during adolescence (Pinto et al., 2010; Silveri et al., 2013). Interneurons in the PFC also undergo protracted maturation throughout adolescence and into adulthood exhibiting increased expression of PV, calcium binding proteins which expand fast-spiking capabilities of GABA neurons to promote neural synchrony and cortical maturation (Fung et al., 2010). Moreover, PNN density increases within the human PFC after pubertal onset (Mauney et al., 2013) and as PNNs primarily enwrap PV neurons, likely contribute to the regulation of both cortical plasticity and PV expression (Carceller et al., 2020). Together, these changes to the GABA system during adolescence increase cortical inhibition to facilitate maturation of PFC functions such as working memory, executive abilities, impulse inhibition, and emotional control (Luna et al., 2004; Silveri et al., 2013; Laube et al., 2020).

The extent to which modifications in the GABA system described above are driven by pubertal changes in ovarian hormones and/or ALLO has yet to be determined. Indeed, the focus of many studies is on how the GABA system and brain function develop over adolescence, which is a more protracted developmental period compared to the shorter timeframe following pubertal onset. Further, many studies do not examine sex as a biological variable thereby limiting what conclusions can be drawn concerning the implications of estradiol, progesterone or ALLO on GABA system activity in young women following pubertal onset.

### Changes to the Gamma-Aminobutyric Acid System Associated With Puberty in Rodents

Pubertal increases in inhibitory tone are also seen in the rodent brain, providing an avenue to study the direct effects of ovarian hormones and ALLO on GABA system activity following pubertal onset in a mammalian system. Rodent studies have shown that the underlying mechanisms that contribute to the development of greater inhibitory tone in the brain following pubertal onset are multifaceted and due in part to increases in GAD expression, as well as increases in the production of inhibitory synapses and GABA transporters (Kilb, 2012; Shen et al., 2020). As described below, growing inhibitory tone following pubertal onset also results from alterations in PV neurons within the medial prefrontal cortex (mPFC, analog to human PFC) and hippocampus (Sisk and Zehr, 2005; Smith et al., 2009; Buzsáki and Wang, 2012; Kilb, 2012; Juraska et al., 2013; Caballero and Tseng, 2016; Zimmermann et al., 2019; Delevich et al., 2021). Inhibitory tone in these regions is further influenced by changes in subunit expression of GABA_A_Rs, which affect GABA_A_R activity (Smith et al., 2009). Evidence suggests that changes to the GABA system within the mPFC and hippocampus of the adolescent female brain may depend on the presence of estradiol, progesterone, and/or ALLO and impact behavior (Sisk and Foster, 2004; Smith et al., 2007; Shen et al., 2010; Juraska et al., 2013; Wu et al., 2014; Caballero and Tseng, 2016; Piekarski et al., 2017a,b).

#### Changes to PV Neurons Associated With Puberty

Expression of PV increases within the rodent mPFC and hippocampus following pubertal onset (Cruz et al., 2003; Kilb, 2012; Caballero et al., 2014; Glausier et al., 2014; Wu et al., 2014; Delevich et al., 2021). In addition, evidence in rodents indicates that both mPFC and hippocampal PV neuronal complexity are enhanced following pubertal onset resulting in more synaptic connections with neighboring neurons over adolescence despite no concomitant increase in PV neuron number (Caballero et al., 2014; Honeycutt et al., 2016; Baker et al., 2017; Shen et al., 2020). During adolescence, PV neurons also begin promoting neural synchrony and the production of gamma oscillations within the mPFC and hippocampus, both of which are key to maturation of cognitive capacity and flexibility as well as emotional regulation (Buzsáki and Wang, 2012; Kilb, 2012). These shifts in PV expression, morphology, and activity enhance inhibitory tone following pubertal onset, positioning PV neurons as pacers of brain maturation during adolescence.

Though PV expression increases within the hippocampus and mPFC of both male and female rats during adolescence (Caballero et al., 2014; Wu et al., 2014; Caballero et al., 2020; Gildawie et al., 2020), rodent studies have shown that gonadal hormones play especially critical roles in promoting PV expression and activity in the female pubertal brain. A study by Wu et al. (2014) showed that ovariectomy (OVX), but not castration, prior to pubertal onset reduces hippocampal PV expression in adult mice (Wu et al., 2014), illustrating the importance of gonadal hormones in organizing patterns of PV expression specifically in the female hippocampus. Activity of PV neurons is also dependent on ovarian hormones. Piekarski et al. (2017a) found that pre-pubertal, but not post-pubertal, OVX in female mice impairs inhibitory signaling in the mPFC (Piekarski et al., 2017a). Additionally, this same study demonstrated that pre-pubertal hormone treatment with estradiol and progesterone accelerates maturation of mPFC inhibitory tone and cognitive development. Together this work suggests that ovarian steroids play a role in pubertal maturation of inhibitory activity within the female mouse mPFC through specific modulation of GABA neurons to affect behavior (Caballero et al., 2014; Piekarski et al., 2017a). Though the type of GABA neuron affected in the Piekarski et al. (2017a) study was not identified, evidence strongly suggests that they were PV neurons due to their patterns of activity, increased activity following puberty, and vulnerability to manipulation of ovarian hormones (Caballero et al., 2014, 2016; Caballero and Tseng, 2016). The effects of ovarian hormones on PV neurons enumerated here are perhaps not surprising, as co-localization of PV neurons and estrogen receptors in the rodent brain (including the hippocampus and mPFC) suggests that estradiol acts directly on PV neurons to influence their activity (Hart et al., 2001; Blurton-Jones and Tuszynski, 2002; Wu et al., 2014; Równiak, 2017). However, increases in both estradiol and progesterone accompany pubertal onset (Piekarski et al., 2017b; Filice et al., 2018; Delevich et al., 2021). Thus, the role of progesterone in PV neuron maturation within the pubertal/adolescent female brain requires further consideration.

In addition to their role in facilitating cognitive maturation during adolescence, mPFC PV neurons also contribute to emotional behaviors specifically in female mice. Following chronic stress during adolescence, PV neuron expression in the mPFC increases, as do some anxiety-like behaviors (Page and Coutellier, 2018). Further, chemogenetic activation of PV neurons within the mPFC was shown to enhance anxiety-like behaviors in adult female, but not male, mice (Page et al., 2019). As adolescence is associated with the emergence of sex differences in mood disorders (Smith et al., 2009), interactions between ovarian hormones and the GABA system during this time may render the female brain exceptionally sensitive to stress and predispose adolescent females to developing anxiety and depression.

Along with increases in both expression and activity of PV neurons, PNN expression also increases following pubertal onset within the mPFC (Drzewiecki et al., 2020). Though this increase is observed in both male and female rats, PNN expression in the mPFC transiently drops at pubertal onset exclusively in females (Drzewiecki et al., 2020), an effect which may serve to facilitate neurite growth and formation of new synapses in the female mPFC. As PNN presence regulates the activity of the PV neurons they surround (Carceller et al., 2020), sex differences in patterns of PNN expression may also reflect modulation of PV neuron activity by ovarian hormones through control of PNN formation. PNN density also increases over adolescence in the human PFC (Mauney et al., 2013) and thus, additional animal research can aid in determining the function of pubertal changes in PNN expression and the underlying hormonal mechanisms.

Together, available evidence suggests a critical role for ovarian hormones in both the expression and function of PV neurons following pubertal onset in females. However, additional investigation of PV neuron development within the female pubertal brain is needed, since many of the studies examining inhibitory maturation during adolescence either do not include female subjects or do not analyze the data by sex. Further, most studies focus on the mPFC or hippocampus and thus it remains to be determined if the pubertal pattern of PNN expression is recapitulated in other brain regions (Caballero et al., 2014, 2020; Caballero and Tseng, 2016). Understanding the role of hormones in maturation of GABA neurons in the developing brain is critical, as activity of these neurons contribute to E/I balance and the neural signatures necessary for the changes in cognitive function and mood associated with adolescent brain development (Piekarski et al., 2017a; Page and Coutellier, 2018; Page et al., 2019).

#### Changes in GABA_A_R Expression Associated With Puberty

In addition to modifications in the expression and activity of PV neurons, pubertal onset induces shifts in GABA_A_R subunit expression, as illustrated in mouse models of puberty (Smith et al., 2009). Changes in the expression of GABA_A_R subunits can affect ion gradients and thus, excitability of the neurons expressing GABA_A_Rs (Smith et al., 2007, 2009; Smith, 2013; Keating et al., 2019).

In the hippocampus of female mice, withdrawal of ALLO at puberty leads to a transient increase in α4βδ GABA_A_R expression on pyramidal neurons to affect behavior (Shen et al., 2007; Smith et al., 2007, 2009; Smith, 2013; Keating et al., 2019). Specifically, pubertal enhancements in hippocampal α4βδ GABA_A_Rs are associated with impairments in hippocampal-dependent learning tasks (Shen et al., 2010). Such impairments in learning appear to be mediated by the δ subunit, the site of neurosteroid action during periods of hormonal flux, and can be reversed by administration of ALLO which reduces tonic inhibition at puberty (Shen et al., 2010). Additional research in mice suggests that pubertal ALLO facilitates spontaneous spiking of pyramidal neurons through reductions of inhibitory tone mediated by hippocampal α4βδ GABA_A_Rs to promote anxiety-like behavior (Shen et al., 2007; Smith, 2013). Together, these data suggest that ALLO *via* its actions at α4βδ GABA_A_Rs affect inhibition during puberty, as well as cognitive and emotional behavior (Shen et al., 2007; Smith et al., 2009; Afroz et al., 2016; Parato et al., 2019).

Pubertal changes in GABA_A_R subunit expression also occur on hippocampal PV neurons (Shen et al., 2020). While expression of δ subunits increases on hippocampal pyramidal neurons, δ subunit expression decreases on hippocampal PV neurons following pubertal onset (Shen et al., 2010, 2020; Keating et al., 2019). Since there are fewer δ subunits on PV neurons following pubertal onset to diminish their activity, these neurons are disinhibited and subsequently promote brain maturation by enhancing inhibitory tone (Smith et al., 2007; Torres-Reveron et al., 2009; Wu et al., 2014; Piekarski et al., 2017b; Shen et al., 2020). Though the behavioral implications of these changes are not yet known, reductions in δ subunit expression on PV neurons may be another mechanism contributing to maturation of cognitive function and emotional regulation during adolescence (Shen et al., 2007, 2010).

In conclusion, existing data point to a role for ovarian hormones and the progesterone metabolite ALLO in the development of inhibition within the female pubertal brain. Continued investigation into the hormonal mechanisms of adolescent brain maturation is important, as the hormonal environment during adolescence influences the trajectory of GABA system development to potentially have long-lasting effects on brain function and behavior (Shen et al., 2007, 2010; Piekarski et al., 2017a,b).

## The Ovarian Cycle

Following pubertal onset, an infradian rhythm known as the ovarian cycle begins. Fluctuations in estradiol, progesterone, and ALLO affect the GABA system to determine inhibitory tone over the ovarian cycle with implications for cycle-related variations in behavior (Maguire and Mody, 2009; MacKenzie and Maguire, 2014).

### Changes to the Gamma-Aminobutyric Acid System Over the Menstrual Cycle

In humans, the menstrual cycle takes place over the course of approximately one month and is comprised of the follicular and luteal phases. Estradiol levels rise during the follicular phase due to the complex interactions of both positive and negative feedback loops within the HPO axis (Oyola and Handa, 2017). The follicular phase ends at ovulation when a mature follicle within the ovaries ruptures and subsequently becomes the corpus luteum, which produces high amounts of progesterone and ALLO during the luteal phase. Estradiol also increases during the luteal phase, though less robustly than during the follicular phase. At menses, estradiol, progesterone, and ALLO levels plummet and remain low for the a few days to mark the beginning of the follicular phase of the following menstrual cycle (Reed and Carr, 2018).

Inhibitory tone changes over the menstrual cycle in association with fluctuations in estradiol, progesterone, and ALLO (Harada et al., 2011; Vigod et al., 2014). Increases in cortical inhibition, gamma oscillation frequency, and neural synchrony are observed during the luteal phase relative to the follicular phase despite a reduction in GABA concentrations (Smith et al., 1999; Epperson et al., 2002; Sumner et al., 2018), while at ovulation GABA concentrations peak within the PFC (De Bondt et al., 2015). Alterations in GABAergic activity and inhibitory tone likely contribute to changes in mood reported across the menstrual cycle associated with specific hormonal profiles, such as increased calmness during the luteal phase and greater sensitivity to psychosocial stress during the follicular phase (Albert et al., 2015; Welz et al., 2016). In contrast, negative changes in mood are observed during the luteal phase in women with premenstrual dysphoric disorder (PMDD). It has been hypothesized that impaired mood regulation in women with PMDD may result from enhanced cortical GABA levels and reduced sensitivity of GABA_A_Rs to fluctuations in ALLO, thereby preventing establishment of typical E/I balance (Epperson et al., 2002; Hantsoo and Epperson, 2020).

### Changes to the Gamma-Aminobutyric Acid System Over the Estrous Cycle

Though similar hormonal shifts occur during the estrous cycle of lab rodents compared to the menstrual cycle of women, there are notable differences in the timing of these shifts which may further depend on species of lab rodent. Rats and mice, the most common models in studies examining neurobiological effects of the ovarian cycle, have 4-6 day estrous cycles although the duration of the cycle may be less consistent in mice (Lovick and Zangrossi, 2021). The estrous cycle consists of four phases: metestrus, diestrus, proestrus, and estrus. Though the names of these phases are the same between rats and mice, the hormonal profiles during these phases differ. In mice, estradiol levels peak during proestrus and progesterone levels peak during diestrus (Wood et al., 2007; McLean et al., 2012; Wu et al., 2013). In rats, peaks in estradiol and progesterone coincide during proestrus (Levine, 2015). ALLO levels also fluctuate across the estrous cycle in relation to progesterone in both rats and mice.

#### Changes in PV and GABAergic Activity Across the Estrous Cycle

Across the estrous cycle, ovarian hormones modify inhibitory tone through PV neuron expression and activity to affect behavior. For example, within the amygdala, PV expression in rats decreases during proestrus, compared to diestrus, contributing to an overall reduction in inhibition (Blume et al., 2017). As the amygdala has been implicated in arousal and stress reactivity, these inhibitory changes across the cycle may explain, at least in part, estrous cycle-related variations in arousal and stress-related behaviors (Lovick and Zangrossi, 2021). Hormonal status can also influence sensory encoding through activity of PV neurons in the barrel cortex of rats, as estradiol enhances both fast spiking interneuron activity (thought to be PV neurons due to their patterns of activity and sensitivity to ovarian hormones) and frequency of inhibitory post-synaptic potentials following social touch during estrus (Clemens et al., 2019). Other work examining PNNs in the medial preoptic area (mPOA), a region of the hypothalamus important for the expression of sexual and parental behaviors, found no changes in PNN number or intensity across the rat estrous cycle (Uriarte et al., 2020). As PNNs preferentially surround PV neurons, these data suggest that though PV neuron expression and activity are responsive to changes in ovarian hormones, plasticity of the neurons may not change with shifting hormones during the estrous cycle. However, these data were derived from separate brain regions and thus it remains unclear the extent to which findings in one region generalize to others (Uriarte et al., 2020).

#### Changes to GABA_A_Rs Over the Estrous Cycle

Ovarian hormones and their metabolites can further modulate inhibitory tone across the estrous cycle through GABA_A_Rs (Löscher et al., 1992; Maguire et al., 2005; Maguire and Mody, 2007, 2009; Lovick, 2012; Wu et al., 2013; Sabaliauskas et al., 2015). Notably, ALLO can act as a PAM at GABA_A_Rs over the estrous cycle to modulate inhibition, an effect that is thought to be mediated by expression of the ALLO-sensitive δ subunit of the GABA_A_R (Maguire et al., 2005; Wu et al., 2013; Sabaliauskas et al., 2015). Expression of this subunit is critically dependent on the cyclic nature of the estrous cycle and ALLO levels, as changes in δ subunit expression do not occur in acyclic mice and are prevented in mice treated with finasteride, a drug which inhibits the metabolism of progesterone to reduce ALLO production (Barth et al., 2014; Wu et al., 2013). These cyclic changes in the expression of neurosteroid-sensitive δ subunit of the GABA_A_R affect neuronal activity and inhibitory tone to influence behavior. For example, enhancements in hippocampal δ subunit expression during late diestrus results in increased tonic inhibition within dentate gyrus granule cells from the female mouse hippocampus, along with reductions in anxiety-like behavior (Maguire et al., 2005; Maguire and Mody, 2007). Changes in δ subunit expression are also seen on inhibitory neurons in the female mouse hippocampus across the estrous cycle (Barth et al., 2014). Specifically, increases in δ subunit expression on PV neurons during diestrus compared to estrus diminish production of gamma oscillations and impair neuronal synchrony within the hippocampus which may have consequence for cognitive function (Barth et al., 2014; Shen et al., 2020). Together, these data show that changes in GABA_A_Rs subunit composition depend on hormonal status and can subsequently affect activity of hippocampal interneurons to modulate inhibitory tone over the estrous cycle. In addition to the hippocampus, other work in rats has examined GABA_A_ receptor subunit composition within the midbrain periaqueductal gray (PAG), a region important for integrating anxiety responses. Within the PAG, expression of α4β1δ GABA_A_ receptors fluctuates over the estrous cycle in relation to changing levels of ALLO to modify GABAergic tone and anxiety-like behavior (Griffiths and Lovick, 2005; Lovick, 2012).

Much of the research examining the effects of shifting ovarian hormones on the GABA system across the estrous cycle has focused on different brain regions and has used different animal model species with variations in hormonal profiles across the ovarian cycle, making direct comparisons across studies difficult. Additionally, most of the existing research addresses the influence of ALLO on the GABA system. Therefore, determining the mechanisms by which estradiol and progesterone affect the GABA system is warranted, as levels of these hormones change over the ovarian cycle and have been shown to affect GABAergic activity and brain functions dependent on proper E/I balance during other hormonal transition periods (Löscher et al., 1992; Maffucci and Gore, 2009; Barth et al., 2014; Blume et al., 2017; Clemens et al., 2019). It is also worth mentioning that there are a limited number of studies which have looked at the effects of hormonal contraceptives on the GABA system and these have found altered cortical GABA concentrations in human hormonal contraceptive users as well as changes in GABA_A_ receptor subunit expression in rodents after prolonged treatment with hormones found in hormonal contraceptives (Follesa et al., 2002; De Bondt et al., 2015). Hormonal contraceptives may give rise to these effects because they contain synthetic estradiol and progesterone analogs that inhibit the HPO axis through negative feedback leading to lower endogenous levels of estradiol and progesterone and a suppression in the fluctuation of ovarian hormones across the cycle as well as a decrease in ALLO (Montoya and Bos, 2017; Porcu et al., 2019). Although hormonal contraceptives are used by millions of women worldwide, their impact on the brain and behavior have not been well studied and warrant greater consideration (Porcu et al., 2019; Taylor et al., 2021).

## Pregnancy and the Postpartum Period

As during puberty and the ovarian cycle, major hormonal shifts occur over the course of pregnancy and the postpartum period (Bloch et al., 2003; De Bonis et al., 2012; Duarte-Guterman et al., 2019). In humans, pregnancy induces profound increases in both plasma estradiol and progesterone, which can reach levels 50- and 10-fold higher, respectively, than at peak concentration during the menstrual cycle due to placental hormone production (Bloch et al., 2003). In rodents, levels of estradiol and progesterone similarly surge during late pregnancy (Concas et al., 1998, 1999; Brunton and Russell, 2010). Likewise, ALLO concentrations rise throughout gestation in rodents and humans reaching peak concentrations during late pregnancy (Concas et al., 1999; Luisi et al., 2000). This accumulation in hormones over the course of pregnancy rapidly declines just prior to (rats) or following (humans) delivery, resulting in a period of hormonal change that affects different aspects of the GABA system and, in turn, inhibitory tone (Concas et al., 1998, 1999; Epperson et al., 2006; Maguire et al., 2009; MacKenzie and Maguire, 2014; Vigod et al., 2014; Maguire, 2019; Deems and Leuner, 2021). As described below, dysregulation in GABAergic signaling has been linked to deficits in maternal care, as well as heightened anxiety- and depression-like behaviors, during the postpartum period.

### Changes in Gamma-Aminobutyric Acid Concentration During Pregnancy and the Postpartum Period in Humans

Central GABA concentrations have been reported to be altered in pregnant and postpartum women. For example, GABA levels in cerebrospinal fluid (CSF) decrease during the last few weeks of pregnancy (Altemus et al., 2004; Vigod et al., 2014) and then significantly increase during labor (Sethuraman et al., 2006). In addition, cortical GABA levels decrease after birth and begin to normalize over the course of the postpartum period (Epperson et al., 2006; Vigod et al., 2014). Though these data do not directly support a causal relationship, they nonetheless suggest that changes in GABA concentrations over late pregnancy and into the postpartum period are related to changes in ovarian hormones and ALLO given their coincident timing.

It has been proposed that aberrant GABAergic activity and a failure to properly maintain E/I balance during the transition into the postpartum period may be a precipitating factor leading to postpartum depression (PPD), a disorder in new mothers characterized by symptoms such as sadness, cognitive impairment, and strained mother-infant interactions (Deligiannidis et al., 2013, 2016; Stewart and Vigod, 2019; Deems and Leuner, 2021). In particular, PPD is thought to arise, at least in part, when there is a failure of GABA_A_Rs to adapt to the abrupt decline in ALLO after birth (Faden and Citrome, 2020; Meltzer-Brody and Kanes, 2020).

### Changes in Inhibition During Pregnancy and the Postpartum Period in Rodents

#### Changes in GABA During Pregnancy and the Postpartum Period in Rodents

GABA concentrations are also altered in the rodent brain during pregnancy and the postpartum period. For example, GABA concentrations significantly decline during late pregnancy in the mouse hippocampus and subsequently normalize following parturition (Smolen et al., 1993). In postpartum rats, CSF concentrations of GABA increase following offspring interaction (Qureshi et al., 1987; Lonstein et al., 2014). GABA modifications during pregnancy and the postpartum period are not limited to GABA concentrations with increases in GABA synthesis (as indicated by higher expression of GAD) reported in the lateral septum of postpartum mice (Zhao et al., 2012) and the mPFC of postpartum rats (Ahmed and Lonstein, 2012; Lonstein et al., 2014). Other work has shown both lower GABA release in the basolateral amygdala of pregnant rats (Young and Cook, 2006) and reduced turnover in the cerebral cortex of late pregnant/early postpartum mice (Smolen et al., 1993), while postpartum rats have both higher basal GABA release and turnover in the mPFC in comparison to virgins (Kornblatt and Grattan, 2000; Arriaga-Avila et al., 2014; Ragan et al., 2022). Overall, these data show complex effects on GABA that are highly dependent on species and brain region. The regions analyzed in these studies have been implicated in behavioral functions that are altered postpartum such as anxiety, fear, maternal care, and maternal aggression, with some work pointing to GABAergic involvement (Lee and Gammie, 2009; Sabihi et al., 2021).

#### Changes in Expression of Perineuronal Nets During Pregnancy and the Postpartum Period in Rodents

Over the course of pregnancy and into the postpartum period, PNN number and intensity change in the rat mPOA, a region that is both critical to establishment of maternal care behaviors and highly plastic during this time (Keyser-Marcus et al., 2001; Duarte-Guterman et al., 2019). Specifically, expression of PNNs in the mPOA steadily increases from mid-gestation to late pregnancy and peaks immediately prior to parturition (Uriarte et al., 2020). PNN expression subsequently drops following parturition, increases again to peak levels one week into the postpartum period, and remains elevated in comparison to cycling rats until weaning (Uriarte et al., 2020). These changes in PNN expression during pregnancy and the postpartum period may be due to extended exposure to ovarian hormones, as induction of hormonal pseudopregnancy recapitulates a similar pattern, though to a lesser extent (Uriarte et al., 2020). Since PNN presence typically limits neuroplasticity (Karetko and Skangiel-Kramska, 2009), changes in PNN expression in the mPOA suggests that PNNs may play a time-dependent role in contributing to both maintenance of established circuitry in this region during pregnancy and transient permission of plasticity to establish the circuitry critical to postpartum maternal behaviors.

Recent data also show that PNN expression in postpartum rats changes in the primary somatosensory cortex (S1) following viral-mediated knockdown of receptors for oxytocin, a neuropeptide important for social bonding and maternal behavior (Grieb et al., 2021). In oxytocin receptor knockdown rats exhibiting disrupted postpartum social and affective behaviors, more plasticity-restricting PNNs were found in the S1 rostral region, and fewer in the S1 caudal region. It is possible that such alterations in PNN expression may disrupt cortical neuroplasticity to affect maternal sensitivity to tactile cues from the young and impact behavior (Grieb et al., 2021). Other work in never-pregnant mice housed in proximity to pups demonstrates that pup interaction, and not hormonal milieu, drive sub-regional differences in PNN density within the somatosensory cortex (Lau et al., 2020; Grieb et al., 2021). Additional studies are needed to better understand PNNs in the maternal brain and the extent to which they contribute to behavior during this time.

#### Changes to GABA_A_Rs During Pregnancy and the Postpartum Period in Rodents

GABA_A_Rs and their capacity to mediate GABAergic inhibition are highly plastic across pregnancy and the postpartum period. Within rat forebrain, GABA_A_R binding affinity is enhanced during late pregnancy and is further enhanced in the postpartum period (Majewska et al., 1989). The brain region(s) driving these effects are unknown but likely do not include the cortex as this region shows a decrease in binding affinity during late pregnancy (Concas et al., 1999). A postpartum reduction in total forebrain GABA_A_R density has also been reported although again, specific brain sites remain to be elucidated (Miller and Lonstein, 2011; Lonstein et al., 2014).

Much of the work examining GABA_A_Rs during pregnancy and the postpartum period has focused on ALLO’s effects on the expression of specific GABA_A_R subunits. Overall this body of work shows that during late pregnancy and the postpartum period, shifts in ALLO lead to changes in subunit composition of GABA_A_Rs to modulate GABA_A_R activity and influence inhibitory tone (Concas et al., 1999; Maguire and Mody, 2008; Licheri et al., 2015). For example, in the hippocampal dentate gyrus of late pregnant rats, high levels of ALLO enhance granule cell expression of the δ subunit to increase tonic inhibition, effects which can be blocked by finasteride treatment (Sanna et al., 2009). Postpartum expression of the δ subunit in the rat hippocampus, as well as tonic inhibition, diminishes following a postpartum decline in ALLO (Smith et al., 1998; Sanna et al., 2009). Though the functional effects of these changes in the rat maternal hippocampus remain to be studied, they likely contribute to the development of behaviors that have been shown to be altered during pregnancy and the postpartum period and that are hippocampal-dependent, such as working memory and anxiety (Pawluski et al., 2006).

Like in rats, female mice show fluctuations in ALLO levels over the course of pregnancy which affects hippocampal δ subunit expression and modulates activity at GABA_A_Rs to influence E/I balance. However, in contrast to rats, expression in mice is instead reduced in the hippocampus as well as in the striatum and thalamus, while remaining stable in the cortex (Maguire et al., 2009). Within 48 hours of delivery, hippocampal δ subunit expression subsequently recovers to virgin levels (Maguire et al., 2009). This pattern of δ subunit expression is critical, as data from mice deficient in the GABA_A_R δ subunit suggest that the inability to regulate δ subunit-containing GABA_A_Rs during pregnancy and the postpartum period results in increased anxiety- and depression-like behaviors, as well as abnormal mothering (Maguire and Mody, 2008; Melón et al., 2018). Importantly, treatment of GABA_A_R δ subunit deficient mice with a synthetic neuroactive steroid GABA_A_R PAM (SGE-516) was shown to decrease PPD-like behaviors and improve maternal care (Melón et al., 2018).

Localized changes in δ subunit expression also occur on hippocampal interneurons during late pregnancy in mice (Maguire and Mody, 2008; Melón et al., 2018). As δ subunit-containing GABA_A_Rs often populate PV neurons in this region and are vulnerable to changes in ALLO, reductions in δ subunit expression during pregnancy in mice lead to a modification of hippocampal PV neuron activity (Ferando and Mody, 2013). Indeed, *in vitro* electrophysiological analyses indicate that the downregulation in δ subunit expression during pregnancy enhances gamma oscillation frequency in hippocampal slices from pregnant mice because of the acute withdrawal from ALLO (Ferando and Mody, 2013). However, application of ALLO at concentrations physiologically equivalent to those seen during pregnancy reduces the frequency of gamma oscillations in the hippocampi of pregnant mice to that observed in virgin mice, suggesting that ALLO acts as a PAM to normalize PV neuron activity during pregnancy through regulation of the δ subunit of GABA_A_Rs (Ferando and Mody, 2013). Following parturition and the subsequent decline in ALLO, δ subunit expression on hippocampal interneurons returns to baseline, as does the frequency of induced gamma oscillations (Ferando and Mody, 2013). This evidence points to modulation of interneuron activity by ALLO at δ subunits, and not changes in interneuron density or connectivity, as main determinants of inhibitory tone in the hippocampus during pregnancy (Ferando and Mody, 2013). Overall, despite differences in mice and rats, these data indicate that high levels of ALLO during pregnancy modulate expression of neurosteroid-sensitive GABA_A_Rs δ subunits to determine E/I balance in the rodent hippocampus to affect behavior.

Other evidence points to modifications in γ subunits of GABA_A_Rs, which are responsive to changing levels of neurosteroids (Maguire and Mody, 2008; Licheri et al., 2015). For example, expression of the γ2L subunit is reduced in the rat hippocampus and cortex, as well as in the mouse hippocampus, with rising ALLO levels during pregnancy which, along with observed changes in expression of the δ subunit, may serve to potentiate inhibitory tone (Concas et al., 1998, 1999; Maguire and Mody, 2008; Maguire et al., 2009; Sanna et al., 2009). Following a postpartum drop in ALLO levels, expression of γ2L in the rat hippocampus and cortex increases to likely facilitate a return of inhibitory tone to a pre-pregnancy state (Concas et al., 1998, 1999; Sanna et al., 2009). In addition to the postpartum changes seen in the hippocampus, γ2 subunit expression increases in the rat paraventricular nucleus of the hypothalamus during lactation (Fénelon and Herbison, 1996), an effect that could be necessary to ensure sufficient stimulation of oxytocin neurons in this region to establish maternal care behaviors (Giovenardi et al., 1997).

Along with δ and γ, α subunit expression also changes over pregnancy and the postpartum period. In rats, hippocampal α4 expression increases during the postpartum period and likely contributes to changes in hippocampal inhibitory tone and anxiety-like behavior (Smith et al., 1998; Sanna et al., 2009). However, some studies report no change in α subunit expression in the rat hippocampus or cortex during pregnancy and the postpartum period (Concas et al., 1998, 1999; Follesa et al., 1998). Thus, despite some heterogeneity, the data overall show cell-, region-, and species-specific changes in δ, γ, and α subunit composition which are important for the establishment of inhibitory tone during pregnancy and the postpartum period.

Together, evidence points to major changes in the GABA system over the course of pregnancy and the postpartum period. While some findings implicate ovarian hormones in these changes, much of the research during pregnancy and the postpartum period to date has focused on ALLO and changes in GABA_A_R subunit composition. There is also work which suggests that another important mediator of inhibition in the pregnant and/or postpartum brain to consider is oxytocin, which aligns with known oxytocin-GABA interactions within the hypothalamus (Giovenardi et al., 1997; Fénelon and Herbison, 2000; Kornblatt and Grattan, 2000; Herbison, 2001; Lonstein et al., 2014) and the mPFC (Sabihi et al., 2014, 2021) to affect maternal, cognitive, and emotional behavior.

## Menopausal Transition and Reproductive Senescence

Aging gives way to reproductive senescence caused by HPO axis disruption (Wise, 1999). In women, the decline in HPO axis activity that characterizes the menopausal transition occurs following ovarian follicle depletion, which in turn promotes production of gonadotropins in an effort to rescue falling concentrations of estradiol, progesterone, and progesterone metabolites (Wise, 1999; Morrison et al., 2006; Andréen et al., 2009; Kimball et al., 2020). Failure of such rescue causes menstrual irregularity and eventually amenorrhea which over time will render an individual reproductively senescent and menopausal, typically around 52 years of age (Jacobs and Goldstein, 2018). Reproductive senescence represents another period of the female lifespan during which hormonal changes impact the GABA system with consequences for declining cognition and mood often experienced during this time (Harden et al., 1999; Andréen et al., 2009; Erel and Guralp, 2011; Jacobs and Goldstein, 2018).

### Changes Within the GABA System During the Menopausal Transition in Women

Like during other periods of hormonal fluctuation, the brain experiences shifts in GABAergic tone during the menopausal transition. Recent work examining pre- and post-menopausal women shows that GABA concentrations in the cortex decline over the menopausal transition and decline further in postmenopausal women with depression (Wang et al., 2016, 2019). Postmenopausal reductions in cortical GABA concentrations may also be relevant to cognitive function, as a recent study shows that hippocampal GABA positively correlates with memory performance in older women (Jiménez-Balado et al., 2021). Other than these few reports, we are unaware of any other work assessing GABAergic activity of postmenopausal women, though decreases in ALLO associated with reproductive senescence suggest that additional changes are likely. As ALLO is both reduced in postmenopausal women (Kimball et al., 2020) and is a PAM of GABA_A_Rs, changes in functional inhibition most likely occur over the course of the menopausal transition (Gordon et al., 2015).

### Models of Reproductive Senescence in Rodents

Comparisons between humans and rodents are difficult in the context of reproductive senescence due to various factors. First, rodents do not undergo menopause but instead as they enter middle age, will experience irregular cycles and eventually will cease ovulation and become acyclic at which time they are either in persistent diestrus or persistent estrus (Kermath and Gore, 2012). Following this transition, which typically begins at approximately 10–12 months of age, estradiol levels depend on if a rodent transitions into persistent diestrus (lower estrogen) or persistent estrus (higher estrogen). This stands in contrast to the to the eventual decline in estradiol observed universally in women (Wise, 1999; Koebele and Bimonte-Nelson, 2016). Second, rodents do not undergo follicular depletion to the same extent as in humans nor do they depend expressly on follicular content within the ovary to determine reproductive senescence (Peng and Huang, 1972; Wise, 1999; Diaz Brinton, 2012). Lastly, many research groups choose to model menopause through OVX, which does not capture the steady decline in hormones that characterize the transition to menopause and thus limits translatability as most women undergo natural, not surgical, menopause (Diaz Brinton, 2012). In fact, many studies modeling menopause through OVX frequently use young rodents, which is inconsistent with the middle-age timing of menopausal transition in women. OVX as a model of menopause is also problematic when studying the GABAergic system, as OVX in rats can contribute to reductions in hippocampal GABA concentrations (Tominaga et al., 2001) and enhances GABA binding across various brain regions dependent on time between OVX and assessment (Bosse and Paolo, 1995; Bossé and Paolo, 1996). Thus, simply reducing ovarian hormones has substantial effects itself on inhibitory tone within the brain. Other work investigating the menopausal transition and reproductive senescence centers on the use of hormone replacement therapy (HRT) to address menopausal symptoms, such as onset of hot flashes and vaginal dryness (Shanmugan and Epperson, 2014; Toffol et al., 2015). This is reiterated in rodent neuroscience research, as the combination of OVX and ovarian hormone supplementation is a commonly used model to study the effects of HRT during menopause on the brain and behavior, including cognition and mood (Dumitriu et al., 2010; Rao et al., 2013; Koebele et al., 2017, 2021; Jacobs and Goldstein, 2018; Prakapenka et al., 2018).

### Changes Within the GABA System During the Transition to Reproductive Senescence in Rodents and in Response to Hormone Replacement Therapy

The effects of HRT on the GABA system have only been examined in a few studies. These studies have shown that supplementation of both estradiol and progesterone following OVX reduces hippocampal GAD expression in rats (Weiland, 1992) and attenuates GABAergic gene expression within the hippocampus and amygdala of rhesus macaques (Noriega et al., 2010). It has also been shown that exogenous hormone administration following OVX in aged female rats ameliorates depressive-like behaviors through a GABAergic mechanism (Rodríguez-Landa et al., 2020), suggesting that HRT may modulate the GABA system to positively affect behavior in reproductively senescent rodents. However, not all studies find beneficial effects of HRT on brain function. One research group used OVX female rats over one year of age to study how supplementation of either progesterone or a synthetic progestin (medroxyprogesterone acetate) commonly used in HRT immediately following OVX affects GAD expression and brain function during reproductive senescence. Using this model, GAD was found to be reduced in the hippocampus and increased in the entorhinal cortex following progesterone or progestin supplementation compared to rats not supplemented with any hormones (Braden et al., 2010). Further, progesterone supplementation following OVX in aged female rats was associated with a decline in working memory, which later studies demonstrated was caused by excessive activity at GABA_A_Rs (Braden et al., 2015). Although other work has shown that HRT can improve working memory, these typically use HRT with estrogenic components suggesting that is important to consider both estrogens and progestins when studying the GABA system during reproductive senescence (Koebele et al., 2021).

It is also worth noting studies that have provided additional insight into how activity of the GABA system within the hypothalamus changes with age (Cashion et al., 2004; Grove-Strawser et al., 2007; Neal-Perry et al., 2008). For example, in middle-aged rats, fluctuations in hypothalamic GAD are attenuated over the course of the estrous cycle resulting in reduced hypothalamic GAD expression compared to adult rats on the day of proestrus (Cashion et al., 2004; Grove-Strawser et al., 2007; Neal-Perry et al., 2008). Estrogen and progesterone receptors may be involved since their expression changes across different regions of the hypothalamus during aging in rats (Wilson et al., 2002; Chakraborty et al., 2003; Kermath and Gore, 2012). Overall, this work suggests possible mechanisms contributing to HPO axis disruption during the transition to reproductive senescence.

Together, these data point to changes occurring in the GABA system during the transition to reproductive senescence in both humans and animals. However, the existing evidence is limited. For example, no studies have examined if and how PV expression or PNNs change over the course of reproductive aging in females. Also lacking are studies looking at GABA_A_ receptor plasticity specifically during the menopausal transition. Furthermore, additional research using animal models that better reflect the steady withdrawal from ovarian hormones are needed to advance understanding of GABAergic changes across the menopausal period as well as in the preceding perimenopausal period. In this regard, the ovarian toxin vinylcyclohexene diepoxide (VCD), which causes a steady decline in ovarian follicles as seen in human menopausal transitions, may be useful (Diaz Brinton, 2012; Koebele and Bimonte-Nelson, 2016; Koebele et al., 2021). Some preliminary work has shown effects of VCD on the brain, though no work to date has examined the GABA system (Koebele et al., 2021). Studies using more translational models of reproductive senescence could provide critical insights into the mechanisms by which changes to the GABA system can impair mood and cognitive function during this critical stage of the female lifespan.

## Targeting GABA Signaling for the Treatment of Mood Dysregulation Across the Female Lifepsan

Recent advances have been made in the treatment of PPD through FDA approval for use of the GABA-modulating drug Brexanolone, a synthetic version of ALLO which stabilizes fluctuations in ALLO levels allowing GABA_A_Rs to steadily adjust. Brexanolone, a PAM of GABA_A_Rs, tempers the dramatic changes in E/I balance that arise postpartum due to ALLO withdrawal and, as a result, has been shown to improve mood, cognition, and mother-infant bonding in women with PPD (Kanes et al., 2017; Dacarett-Galeano and Diao, 2019; Faden and Citrome, 2020; Meltzer-Brody and Kanes, 2020). The success of Brexanolone in treating PPD adds further credence to GABAergic dysfunction and withdrawal from ALLO as contributing factors involved in the pathophysiology of PPD. While Brexanolone must be intravenously administered in a hospital setting, recent data suggest another neuroactive steroid GABA_A_R PAM drug, Zuranolone, is also effective in reducing PPD symptoms and unlike Brexanolone, can be orally administered (Deligiannidis et al., 2021). Additional GABA-modulating drugs are also under investigation for the treatment of mood dysregulation associated with other times of hormonal change including PMDD (Bixo et al., 2018; Bäckström et al., 2021; Schweizer-Schubert et al., 2021) and menopausal depression (Saripalli et al., 2021; Schweizer-Schubert et al., 2021) but more data are needed.

## Conclusion

**FIGURE 2:**
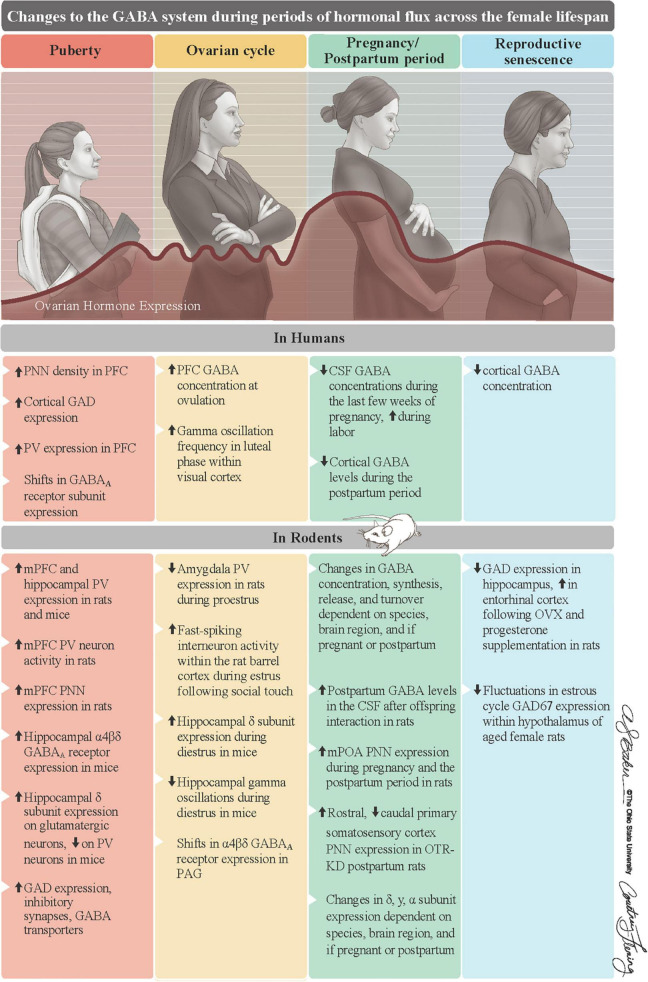
*Summary of changes to the GABA system of females during periods of hormonal flux.* Ovarian hormones substantially change across the female lifespan. They first increase at puberty, fluctuate on an infradian cycle during reproductive years, surge to their lifelong peak during pregnancy, and steadily decline into reproductive senescence. Along with ALLO, these fluctuations in ovarian hormones, which are mostly recapitulated in rodents, are associated with changes to GABA system in both humans (top row) and rodents (bottom row). Reproduced with the permission of The Ohio State University, patterned after Figure 1 in Barrientos et al. (2019). ALLO = allopregnanolone, CSF = cerebrospinal fluid, GAD = glutamate decarboxylase, OTR-KD = oxytocin receptor knockdown, mPFC = medial prefrontal cortex, mPOA = medial preoptic area, PAG = periaqueductal gray, PNNs = perineuronal nets, PV = parvalbumin.

The ovarian hormones estradiol and progesterone, as well as the progesterone metabolite ALLO, exert substantial influence over the GABA system during periods of hormonal flux that characterize the female lifespan (Figure 2). However, there remain gaps in our understanding of the GABA system during these hormonally dynamic periods. This is in part because our current knowledge is based on results that often span different brain regions or focus on only one aspect of the GABA system. There are also components of the GABA system that remain almost completely unexplored. For example, PV neurons have been the focus but are not the sole GABA neurons in the brain. Thus, whether other GABA neurons, like SST and VIP neurons, are affected in relation to hormonal fluctuations across the female lifespan should be examined. In addition, the role of GABA_B_ and GABA_C_ receptors in modulating inhibitory tone during periods of hormonal transition periods warrants exploration, as some evidence suggests that GABA binding capacity at GABA_B_ receptors differ between stages of the estrous cycle (Al-Dahan et al., 1994). It is also important to consider that different types of estrogen predominate during different phases of the female lifespan, yet the focus of ongoing research centers on estradiol, the most common type in women of child-bearing age (Stillwell, 2016). For example, minimal research has examined the effects of estriol, the main estrogen during pregnancy (Falah et al., 2015), and estrone, the predominant form of estrogen during menopause (Cui et al., 2013), on the GABA system. Further insight into how hormones affect the GABA system will broaden our understanding of the female brain and could lead to better treatments for women experiencing changes in brain function at each of these hormonal transition periods.

## Author Contributions

Both authors contributed to the article and approved the submitted version.

## Conflict of Interest

The authors declare that the research was conducted in the absence of any commercial or financial relationships that could be construed as a potential conflict of interest.

## Publisher’s Note

All claims expressed in this article are solely those of the authors and do not necessarily represent those of their affiliated organizations, or those of the publisher, the editors and the reviewers. Any product that may be evaluated in this article, or claim that may be made by its manufacturer, is not guaranteed or endorsed by the publisher.

## Abbreviations

ALLO: allopregnanolone, 3α-Hydroxy-5α-pregnan-20-one, 3α,5α-Tetrahydroprogesterone, or 3α,5α-THP
CSF: cerebrospinal fluid
Estradiol: 17β-estradiol
E/I: excitatory/inhibitory
GABA: gamma-Aminobutyric acid
GABA_A_R: GABA_A_ receptor
GAD: glutamate decarboxylase
HPO: hypothalamic-pituitary-ovarian
HRT: hormone replacement therapy
mPFC: medial prefrontal cortex
mPOA: medial preoptic area
OVX: ovariectomy
PAM: positive allosteric modulator
PFC: prefrontal cortex
PNNs: perineuronal nets
PPD: postpartum depression
PV: parvalbumin
SST: somatostatin
VCD: vinylcyclohexene diepoxide
VGAT: vesicular GABA transporter
VIP: vasointestinal peptide.
